# Fecal Implants From *App*^*NL*–*G*–*F*^ and *App*^*NL*–*G*–*F*/*E*4^ Donor Mice Sufficient to Induce Behavioral Phenotypes in Germ-Free Mice

**DOI:** 10.3389/fnbeh.2022.791128

**Published:** 2022-02-08

**Authors:** Payel Kundu, Keaton Stagaman, Kristin Kasschau, Sarah Holden, Natalia Shulzhenko, Thomas J. Sharpton, Jacob Raber

**Affiliations:** ^1^Department of Behavioral Neuroscience, Oregon Health & Science University, Portland, OR, United States; ^2^Department of Microbiology, Oregon State University, Corvallis, OR, United States; ^3^Carlson College of Veterinary Medicine, Oregon State University, Corvallis, OR, United States; ^4^Department of Statistics, Oregon State University, Corvallis, OR, United States; ^5^Division of Neuroscience ONPRC, Department of Neurology, Psychiatry, and Radiation Medicine, Oregon Health & Science University, Portland, OR, United States; ^6^College of Pharmacy, Oregon State University, Corvallis, OR, United States

**Keywords:** germ-free mice, fecal implants, behavioral testing, biosafety cabinet, APP, APOE, gut microbiome, cortical Aβ

## Abstract

The gut microbiome and the gut brain axis are potential determinants of Alzheimer’s disease (AD) etiology or severity and gut microbiota might coordinate with the gut-brain axis to regulate behavioral phenotypes in AD mouse models. Using 6-month-old human amyloid precursor protein (hAPP) knock-in (KI) mice, which contain the Swedish and Iberian mutations [APP NL-F (*App*^NL–F^)] or the Arctic mutation as third mutation [APP NL-G-F (*App*^NL–G–F^)], behavioral and cognitive performance is associated with the gut microbiome and APP genotype modulates this association. In this study, we determined the feasibility of behavioral testing of mice in a biosafety cabinet and whether stool from 6-month-old *App*^NL–G–F^ mice or *App*^NL–G–F^ crossed with human apoE4 targeted replacement mice is sufficient to induce behavioral phenotypes in 4-5 month-old germ-free C57BL/6J mice 4 weeks following inoculation. We also compared the behavioral phenotypes of the recipient mice with that of the donor mice. Finally, we assessed cortical Aβ levels and analyzed the gut microbiome in the recipient mice. These results show that it is feasible to behaviorally test germ-free mice inside a biosafety cabinet. However, the host genotype was critical in modulating the pattern of induced behavioral phenotypes as compared to those seen in the genotype- and sex-match donor mice. Male mice that received stool from *App*^NL–G–F^ and *App*^NL–G–F/E4^ donor genotypes tended to have lower body weight as compared to wild type, an effect not observed among donor mice. Additionally, *App*^NL–G–F/E4^ recipient males, but not females, showed impaired object recognition. Insoluble Aβ40 levels were detected in *App*^NL–G–F^ and *App*^NL–G–F/E4^ recipient mice. Recipients of *App*^NL–G–F,^ but not *App*^NL–G–F/E4^, donor mice carried cortical insoluble Aβ40 levels that positively correlated with activity levels on the first and second day of open field testing. For recipient mice, the interaction between donor genotype and several behavioral scores predicted gut microbiome alpha-diversity. Similarly, two behavioral performance scores predicted microbiome composition in recipient mice, but this association was dependent on the donor genotype. These data suggest that genotypes of the donor and recipient might need to be considered for developing novel therapeutic strategies targeting the gut microbiome in AD and other neurodegenerative disorders.

## Introduction

Alzheimer’s disease (AD) is the most common form of dementia accounting for 60-80% of dementia cases worldwide (The Alzheimer’s Association, 2021). As the percentage of the population above 65 years old steadily increases, so does the prevalence of this devastating disease. Early intervention before pathology becomes advanced seems critical. Most therapies for AD to date have targeted the processing of amyloid precursor protein (APP) or amyloid beta (Aβ) but have met with limited success. For example, there was a recent FDA approval for using antibodies to clear Aβ in AD patients; however, there are no convincing beneficial cognitive effects yet (Selkoe, 2021). Thus, new avenues for treatment are much needed.

Gut microbiota can coordinate with the gut-brain axis to regulate motor impairments and neuroinflammation in a mouse model of Parkinson’s disease (PD) (Sampson et al., 2016). For example, challenging 3-month-old mice expressing α-synuclein (aSyn) with the A53 mutation with dextran sulfate sodium (DSS), resulted in invasion of macrophages in the lining of the gut wall and greater behavioral impairments, brain pathology, and neuroinflammation both 3 (Kishimoto et al., 2019) and 21 months after exposure (Gratwohl and Al, 2018; ALZFORUM, 2019). Gut microbiota dictate the physiological response to DSS (Ding et al., 2019), which can drive subsequent changes to the microbiome and measures of anxiety in C57BL/6J mice (Nyuki et al., 2018; Wei et al., 2019). In a mouse neurotoxin model of PD, gut microbiome α-diversity was associated with sensorimotor function measured by a rotarod task (Torres et al., 2018). Microbiome composition was also associated with cued fear learning (Torres et al., 2018). In addition, antibiotic treatment ameliorated, while microbial re-colonization promoted, the development of PD pathophysiology in adult animals (Sampson et al., 2016). Additionally, fecal transplants from PD patients compared to healthy human donors exacerbated impairment in α-synuclein-overexpressing mice (Sampson et al., 2016). These results suggest that specific taxa of bacteria in the gut could modulate aspects of behavior and cognition within the context of neurodegenerative disease.

The gut microbiome and the gut brain axis are potential determinants of AD etiology or severity and gut microbiota might also coordinate with the gut-brain axis to regulate behavioral phenotypes in AD mouse models. Using 6-month-old human amyloid precursor protein (hAPP) knock-in (KI) mice (Saito et al., 2014, 2016), which contain the Swedish and Iberian mutations [APP NL-F (*App*^NL–F^)] or the Arctic mutation included as a third mutation [APP NL-G-F (*App*^NL–G–F^)], we previously showed that behavioral and cognitive performance is associated with the gut microbiome and that APP genotype modulates this association (Kundu et al., 2021). This model avoids the overexpression of human APP alongside mouse APP, a confounding feature often present in human APP transgenic mouse lines; in the mouse model used in this study, hAPP is expressed at wild-type levels and no mouse APP is expressed. Hence, this model yields elevated levels of pathogenic amyloid beta (Aβ) and associated neuroinflammation (Saito et al., 2014). The gut microbiome links to hippocampal DNA methylation in these mice. A 1 Kb region overlapping the 3′UTR of the *Tomm40* gene and the promoter region of the *Apoe* gene – both genes that modulate AD risk (Roses et al., 2016) – were more methylated in the hippocampus of *App*^NL–G–F^ than WT mice. Despite these observed links between human APP genotype, behavior, and the gut microbiome, no study has yet determined if the microbiome mediates the effect of APP genotype on behavior outcomes.

To assure that the gut microbiome of the recipient mice is maintained, it is important to only open the cage in a biosafety cabinet. In this study, we determined the feasibility of behavioral testing of mice in a biosafety cabinet and whether stool from 6-month-old *App*^NL–G–F^ mice or *App*^NL–G–F^ crossed with human apoE4 targeted replacement mice is sufficient to induce behavioral phenotypes in 4-5 month-old germ-free C57BL/6J mice 4 weeks following inoculation. In an experiment to establish the conditions under which stable microbial populations can be established in germ-free mice, a review of data generated at five research institutions found that stability was reached in all colonies after 3 weeks (Eberl et al., 2020). Therefore, we included a 4-week incubation period to account for that stabilization period. In the current study, the age range of mice at behavioral testing was 4.00-5.27 months of age (mean age: 4.34 months). We recognize that this age range is slightly younger than 6 months of age. We did not wait 6 months in this first proof of concept study as there is a risk of maintaining the gut microbiome over longer time outside a microisolator. ApoE4 is the biggest genetic risk factor for developing late-onset Alzheimer’s disease and we hypothesized that adding human apoE4 would worsen the cognitive impairment seen in *App*^NL–G–F^ mice at 6 months of age. We recently found that *App*^NL–G–F/E4^ mice develop impairments in fear learning at 6 months of age. To determine the role of the host genotype in the behavioral phenotypes, we also compared the behavioral phenotypes of the recipient mice with that of the donor mice. In addition, we analyzed soluble and insoluble cortical Aβ40 and Aβ42 levels and the gut microbiome in the recipient mice and assessed whether they related to the behavioral measures.

## Materials and Methods

### Animals and Sample Collection

Fecal boli were collected from 6-month-old *App*^NL–G–F^, *App*^NL–G–F/E4^, and wild-type (WT) mice on a C57BL/6J background. All donor mice were bred in the same colony room at OHSU. The 6-month time point was selected as mice with the *App*^NL–G–F^ mutation develop behavioral and cognitive phenotypes at this time point (Saito et al., 2014, 2016). For mice of each genotype, the fecal boli were pooled from males and females separately. The number of fecal boli donor mice from for each genotype and sex was as follows: 3 *App*^NL–G–F^ females, 3 *App*^NL–G–F^ males, 10 *App*^NL–G–F/E4^ females, 13 *App*^NL–G–F/E4^ males, 6 WT females, and 4 WT males. Recipient mice were genotype- and sex-matched to donor mice. The recipient mice were 8-9-week-old wild-type C57BL/6J germ-free mice, with the following group sizes: *App*^NL–G–F^ microbiome recipients (*n* = 9 females, 10 males*), App^NL–G–F/E4^* microbiome recipient (*n* = 11 females, 12 males), WT microbiome recipients (*n* = 10 females, 8 males). The sample size was calculated with a desired power level of 80% and anticipated differences in group means and standard deviations informed by our previous work with human APP-mutant KI mice.

We designed a fecal transplant protocol to lead to a stable colonization of germ-free mice. Based on previous literature, a total of 225 mg of fecal material administered over several gavage sessions was sufficient to establish a lasting change in the gut microbiome^1–3^. In this study, mice were orally gavaged with 0.2 ml of a frozen fecal suspension (1 g fecal material suspended in 5 ml of a 1% cysteine-containing PBS solution), twice a week for three consecutive weeks and for a total of six times. Thus, mice received each a total of 240 g of fecal material. For colonization, mice were moved from germfree isolators to static sterile cages and manipulated in a biosafety cabinet as described for the “out-of-the-isolator” gnotobiotics (Faith et al., 2014). After 3-4 weeks, mice were shipped from Oregon State University in sealed HEPA-filtered cages (Innovive) to Oregon Health and Science University (OHSU). At OHSU, the mice were maintained in sealed HEPA-filtered cages on a Thoren ventilated rack, given *ad libidum* access to sterile water and standard mouse chow (LabDiet 5L0D) and handled in a BL-2 hood, including during behavioral and cognitive testing, in a designated room in the animal facility not housing any other mice. All recipient mice were housed and tested in the same room and building on the OHSU campus. All donor mice were housed and tested in a separate room and building from the recipient mice on the OHSU campus. Recipient and donor mice were both kept in HEPA filtered cages on ventilated racks of the same type and manufacturer. Testing was conducted in three cohorts of 17-24 mice each. For the duration of the study, mice were housed in groups of 2-5 with other mice from the same genotype. Lights were kept on a 12 h light: 12 h dark cycle. The recipient mice were approximately 4.5 months of age at testing.

We also compared the behavioral and cognitive performance of the three genotypes of 6-month-old donor *App*^NL–G–F^, *App*^NL–G–F/E4^ and WT mice. The behavioral and cognitive performance of 6-month-old *App*^NL–G–F^ and WT mice was recently reported^4^. The sample sizes for the behavioral and cognitive testing of the donor mice for were as follows: *App*^NL–G–F^ (*n* = 13 females, 14 males), *App*^NL–G–F/E4^ (*n* = 10 females, 13 males), and WT (*n* = 11 females, 11 males) mice.

### Behavioral and Cognitive Testing

Behavioral and cognitive testing was conducted and the data analyzed by an experimenter blinded to the genotype of the mice. The group code was broken once the data was analyzed. The test battery of the recipient mice included tests used previously in our lab to characterize cognition in the donor mice^4^ and the spatial version of the Y maze with 24 h between training and testing described below. In most cases, one behavioral test was conducted per day, except for the spontaneous alternation Y-maze and the wire hang. Those tests were conducted on the same day, separated by 4 h. Procedures complied with the NIH Guide for the Care and Use of Laboratory Animals and with IACUC approval at OHSU. Behavioral and cognitive testing of recipient mice was conducted in the following order: spontaneous alternation in the Y-maze, performance in the wire hang test, measures of activity and anxiety in the open field, novel object recognition, and spatial version of the Y maze with 24 h between training and testing. Between trials of each test in the recipient mice, all testing surfaces were cleaned with a 70% ethanol solution to eliminate odor cues and decrease the chance of microbiome cross-transfer in the case of recipient mice. As the recipient mice were tested in a biosafety cabinet, some adjustments were made to ensure that equipment could fit and was readily operable. For each test below, testing specifications are indicated separately for recipient and donor mouse testing when different.

### Body Weights

In recipient mice, body weights were assessed at the beginning and end of the behavioral testing. There were no differences in body weights between those two time points. Mean body weight for each mouse was used for the analyses.

### Y Maze

Activity levels and hippocampus-dependent spontaneous alternations were assessed in the Y-maze as previously described^4^. For recipient mice, the Y-shaped maze (Harvard Apparatus, Panlab, Holliston, MA, United States) was smaller than the one used for donor mice and had raised sides and was made of non-reflective opaque gray plastic (30 cm × 6 cm × 15 cm). For donor mice, the Y-shaped maze (O’ Hara & Co., Ltd., Tokyo, Japan) had raised sides (3.8 cm bottom width, 12.55 cm top width, 12.55 cm height) with plastic, opaque gray arms (37.98 cm length). Mice were placed in the center of the maze at the beginning of a 5-minute trial. Performance of the mice was tracked using Ethovision 14 XT video tracking software (Wageningen, Netherlands). Digital videos were later analyzed to measure the number of arm entries and to calculate the percent spontaneous alternations. The criteria for an arm entry was when all four limbs were within the arm. The spontaneous alternation percentage was calculated by dividing the number of 3-arm alternations by the number of possible 3-arm alternations and multiplying the value by 100.

### Wire Hang

The wire hang was performed as previously described by van Putten (2011). A combination of motor function, balance, endurance, and muscle strength was assessed using the wire hang task, adopting the “falls and reaches” method. While the apparatus was placed in the biosafety cabinet, it was otherwise the same for recipient and donor mice. Mice were placed on a 2 mm metal wire suspended 35 cm above soft bedding so that they were hanging only by their front paws. The wire was suspended between two vertical metal posts. Initial placement onto the wire was with the forepaws only, though once the trial began, use of back paws was also allowed. Mice started with a “fall score” of 10 and a “reach score” of 0. Over the duration of 180s, mice lost 1 point from the score every time they fell and gained 1 point every time they reached one of the poles holding up the wire. The time of each fall or reach event was also recorded. Each time a mouse fell or reached, the timer was paused to place the mouse again on the center of the wire.

### Open Field and Novel Object Recognition

Exploratory activity and anxiety were measured in the open field. In this task, enclosures with slightly different dimensions were used for the recipient (L 39.37 cm × W 39.37 cm × H 39.37 cm) and donor (L 40.6 cm × W 40.6 cm × H 40.6 cm) mice. For the recipient mice, collapsible enclosures were used to fit inside the biosafety cabinet. Recipient mice were placed into a well-lit arena (366 lux) for a 5-min trial on 2 consecutive days. The following day, two identical objects were placed in the open field and mice were allowed to explore for a 15-min trial. The objects were placed 10 cm apart and 15 cm from the adjacent walls of the arena. The next day, one object was replaced with a novel object and mice were allowed to explore the open field for 15 min. During object recognition trials, the objects were affixed to the floor of the arena using masking tape. Physical interaction with the object in the form of sniffing within a 2 cm proximity was coded as object exploration. Performance of mice was tracked using Ethovision 14 XT video tracking software. Time spent in the center of the open field was analyzed to assess measures of anxiety. The center zone was 19.77cm on each side. Videos were later hand scored to analyze object exploration.

### Spatial Y Maze

In recipient mice only, the spatial Y-maze test was conducted using the same apparatus as the one used in the Y-maze task above for the recipient mice (Harvard Apparatus, Panlab, Holliston, MA, United States). This task was conducted over 2 consecutive days. On day 1, one arm was blocked off and mice were allowed to explore the maze for 15 min. Extra-maze spatial cues were taped on all three walls of the biosafety cabinet in which the mice were being tested. On day 2, all of the arms were accessible, and mice were allowed to explore for a 5-min trial. Performance of the mice was tracked using Ethovision 14 XT software. Digital videos of day 2 were later analyzed to measure the number of entries into and the percent time spent in the novel arm (the arm that was blocked off during day 1). The criteria for an arm entry was when all four limbs were within the arm.

### Cortical Aβ ELISAs

Cortices of *App*^NL–G–F^ and *App*^NL–G–F/E4^ donor and recipient mice were processed for analyses of soluble and insoluble Aβ40 and Aβ42 levels using Invitrogen ELISA kits (catalog numbers KHB3481 and KHB3441m, respectively; ThermoFischer Scientific, Waltham, MA, United States), according to the recommended guidelines in the production information sheets. To a thawed cortical tissue sample (both hemispheres), 400 μl of buffer A (phosphate-buffered saline containing a protease inhibitor tablet (cOmpleteTM, 11836170001 Roche, Millipore Sigma, Burlington, MA, United States and filtered before use) was added. The tissue was homogenized using a Polytron for 10 s and subsequent a sonicator, and centrifuged at 45,000 rpm for 20 min at 4°C. The supernatant was collected as the soluble fraction. The same volume of buffer A was used to loosen the pellet. The sample was centrifuged again at 45,000 rpm for 5 min at 4°C. After removing the supernatant in a separate tube. the pellet was dissolved in Buffer B (containing 6 M Guanidine H-Cl and 50 mM Tris and filtered before use) and incubated at room temperature for 1 h. After this incubation, the sample was sonicated for 20 s and the extracted pellet was centrifuged at 45,000 rpm for 20 min at 4°C. The supernatant was collected as the insoluble fraction. Pilot experiments were performed to determine the optimal sample dilution. For the analyses of insoluble Aβ levels, the tissue samples were diluted 1: 4,000. For analysis of the insoluble Aβ levels, undiluted tissues samples were used. Standard curves were generated with the same buffer dilution as the samples. The ELISAs were read at 450 nm using a SpectraMax iD5 Multi-Mode Microplate Reader (Molecular Devices, VWR 76175-474, San Jose, CA, United States). The standard curves were generated and the levels in the samples determine using GraphPad Prism software, San Diego, CA, United States). Total protein amounts in the samples were determined by BCA protein assay kit (Pierce, Thermo Scientific, catalog #23225, Waltham, MA, United States) and reading the samples at 562 nm using the iD5 Reader.

### Gut Microbiome Analyses

To assesse the microbiome of all animals, we used a sterile technique to collect fecal boli samples during the first day of object recognition testing and stored samples at −80°C until analysis. 16S rRNA gene sequence libraries were prepared using standard procedures (Walters et al., 2015) as described (Torres et al., 2018; Raber et al., 2019). Bacterial 16S rDNA sequences were PCR amplified and sequenced as described (Gaulke et al., 2016; Torres et al., 2018; Raber et al., 2019). Briefly, DNA was extracted from collected fecal pellets using the QIAamp PowerFecal DNA kit (Qiagen, Hilden, Germany) and the V4 region of the 16S rDNA gene amplified in triplicate using the Earth Microbiome Project 16S PCR protocol. PCR reactions were cleaned using the UltraClean PCR clean-up kit (Qiagen, Hilden, Germany) and samples were diluted to 200 ng of DNA per sample. The prepared libraries were submitted to the Oregon State University Center for Quantitative Life Sciences (CQLS) for 250 bp paired-end sequencing on an Illumina MiSeq instrument. Quality control, exact sequence variants clustering, and chimera removal were conducted using the dada2 package (Callahan et al., 2016) for R (R Core Team, 2017). Standard approaches were used to quality control 16S sequences and resolve amplicon sequence variants (ASVs) using DADA2 (Callahan et al., 2016).

### Statistical Analysis

Data are expressed as mean ± SEM. Behavioral and cognitive performance measures for were analyzed using ANOVAs or repeated-measures ANOVAs. In the case of repeated-measures ANOVA, sphericity was tested and Greenhouse–Geisser corrections were used when appropriate. For the percent time spent exploring the novel and familiar objects, paired *t*-tests were used. Statistical significance was determined using an error probability level of *p* < 0.05. As we *a priori* expected sex-dependent effects, the behavioral and cognitive data were analyzed separately in females and males with donor genotype as a fixed factor. When appropriate, a Dunnett’s *post hoc* test was conducted with the WT genotype as the set comparison. Data were analyzed using SPSS Statistics for Windows (Version 25, Armonk, NY: IBM Corp., Chicago, IL, United States) and Graphpad Prism software (San Diego, CA, United States).

The microbiome sequence data analyses methods were conducted in *R* (R Core Team, 2017). Prior to biodiversity (alpha-diversity) analyses, ASV total abundances in each sample were rarefied (resampled with replacement) to 20,407 counts. In order to assess the relationship between the biodiversity of the microbiome and mouse behavioral test scores, we computed four alpha-diversity metrics for all samples: Observed ASVs (*i.e.*, richness), Chao1 index, Shannon index, and Simpson index. The association between these metrics and individual covariates was quantified using linear regression. Prior to microbiome composition (beta-diversity) analyses, we applied a centered log-ratio (CLR) transformation on the raw ASV counts in order to account for the fact that microbiome data is inherently compositional and to avoid spurious associations caused by analyzing the raw counts of such data (Gloor et al., 2017). To assess the relationship between the composition of the microbiome and mouse behavioral test scores, we computed the Aitchison distance (Euclidean distance on CLR-transformed counts) for all samples. Permutation analysis of variance (PERMANOVA) statistically quantified the association between compositional distance and mouse behavior. We also assessed the significance of association between the abundance of individual microbial taxa and both mouse behavioral scores and genotype using compound Poisson generalized linear regression (Sharpton et al., 2017) to appropriately model the over-dispersion and zero-inflation often observed in microbiome count data.

## Results

For an overview of the behavioral phenotypes in recipient and donor mice, please see Table 1.

**TABLE 1:** A summary table indicating the main effects on body weights and behavioral performance in donor and recipient mice, separated by sex^1^.

Phenotype	Body weight (g)	Y-maze	Wire hang	Open field
**Females**				
Donor	*App^NL–G–F/E4^* lighter than WT	*App*^NL–G–F^ had less spontaneous alternation than WT; *App*^NL–G–F^ and *App^NL–G–F/E4^* had less arm entries than WT	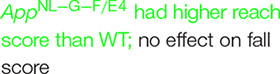	No effect on activity, no effect on time spent in center
Recipient	*App^NL–G–F/E4^* heavier than WT	No effect on arm entries	No effect on reach score, no effect on fall score	No effect on activity, no effect on time spent in center
**Males**				
Donor	No effect	No effect in spontaneous alternation; no effect on arm entries	*App*^NL–G–F^ had lower reach score than WT; no effect on fall score	*App^NL–G–F/E4^* less active than WT;
Recipient	*App*^NL–G–F^ and *App^NL–G–F/E4^* lighter than WT	No effect in spontaneous alternation, no effect on arm entries	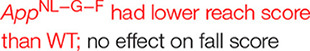	No effect on activity; no effect on time spent in center

*^1^Red indicates a decreased value in App^NL–G–F^ or App^NL–G–F/E4^ mice compared to wild-type, and green indicates an increased value in App^NL–G–F^ or App^NL–G–F/E4^ mice compared to wild-type. In recipient mice, App^NL–G–F^ or App^NL–G–F/E4^ refers to the genotype of the mice they received fecal transplants from.*

### Body Weights

**FIGURE 1:**
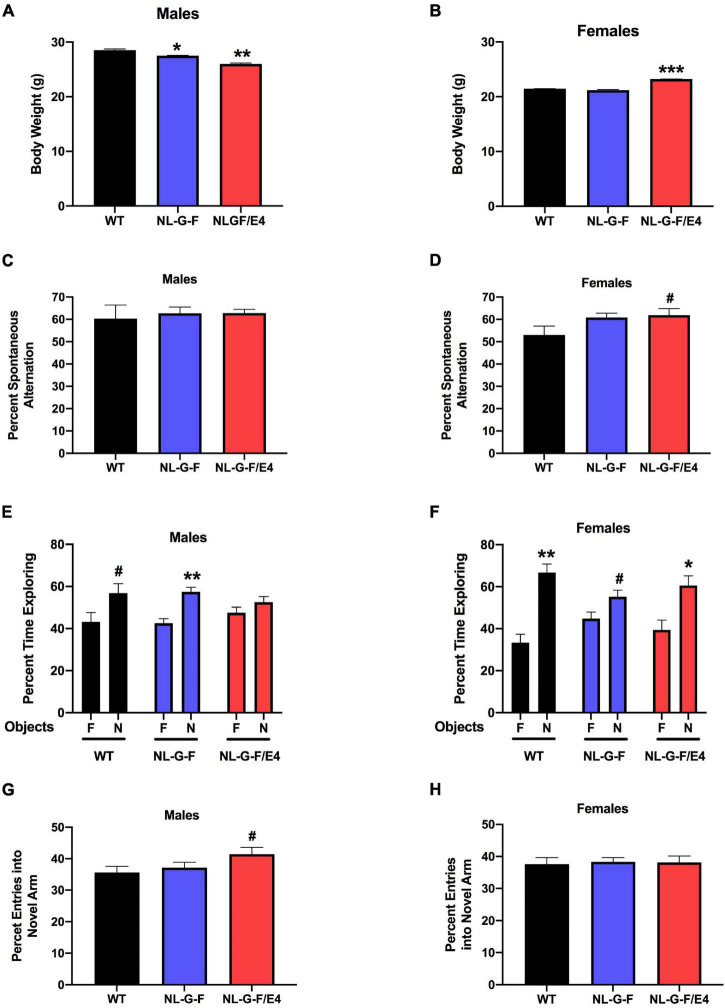
**(A)** In recipient mice, there was an effect of genotype on body weights of males (*F*(2,27) = 96.50, *p* = 0.0022). *App*^NL–G–F^ and *App*^NL–G–F/E4^, males were lighter than WT males. *p = 0.0219; **p = 0.0016. **(B)** In recipient mice, there was an effect of genotype on body weights of females (*F*(2,27) = 179.5, *p* = 0.0008). *App*^NL–G–F/E4^ females were heavier than WT females. ***p = 0.0010, Dunnett’s). **(C)** There was no effect of genotype on spontaneous alternation in male recipient mice. **(D)** There was a trend toward a higher percent spontaneous alternation in *App*^NL–G–F/E4^ than WT females. ^#^*p* = 0.09, Dunnett’s. **(E)** There was a trend toward preferential exploration of the novel object in WT males, preferential exploration of the novel object in *App*^NL–G–F/E4^ males, but *App*^NL–G–F^ males showed impaired object recognition. **p = 0.0037; ^#^*p* = 0.0854; (*t*-tests). **(F)** In female recipient mice, WT [*t* = 4.094, (*t*-test)] and *App*^NL–G–F/E4^ [*t* = 2.278, (*t*-test)] mice spent more time exploring the novel than familiar object and there was a trend toward *App*^NL–G–F^ mice exploring the novel object more than the familiar one. **p = 0.0014; *p = 0.0230; ^#^*p* = 0.0681 (*t*-tests). **(G)** There was a trend toward a higher percent entries of *App*^NL–G–F/E4^ than WT mice into the novel arm of the Y maze. ^#^*p* = 0.0985 (Dunnett’s). **(H)** There was no effect of genotype on the percent entries into the novel arm in the Y maze in recipient female mice.

**FIGURE 2:**
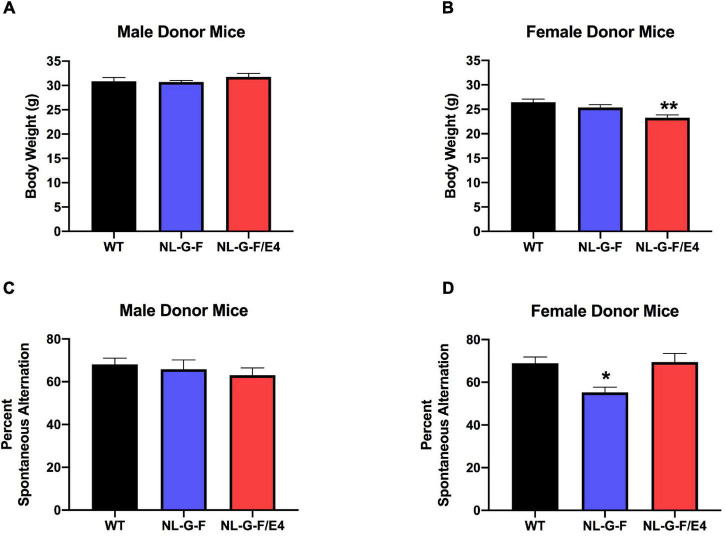
**(A)** There was no effect of genotype on body weights of male donor mice. **(B)** There was an effect of genotype on body weights of female donor mice (*F*(2,34) = 6.044, *p* = 0.0057). *App*^NL–G–F/E4^ females were lighter than WT females. **p = 0.0032, Dunnett’s. **(C)** There was no effect of genotype on spontaneous alternation in male donor mice. **(D)** There was an effect of genotype on spontaneous alternation in female donor mice (*F*(2,40) = 6.136, *p* = 0.0047). The percent spontaneous alternation was lower in *App*^NL–G–F^ than WT female donor mice. *p = 0.0163, Dunnett’s.

In recipient mice, there was an effect of genotype on body weights of males (*F* (2, 27) = 96.50, *p* = 0.0022) and females (*F* (2, 27) = 179.5, *p* = 0.0008). *App*^NL–G–F^ (*p* = 0.0219, Dunnett’s) and *App*^NL–G–F/E4^ (*p* = 0.0016, Dunnett’s) males were lighter than WT males (Figure 1A). In contrast to males, in females *App*^NL–G–F/E4^ females were heavier than WT females (*p* = 0.0010, Dunnett’s) (Figure 1B). In contrast to male recipient mice, there was no effect of genotype on body weights of male donor mice (Figure 2A). There was an effect of genotype on body weights of female donor mice (*F* (2, 34) = 6.044, *p* = 0.0057) (Figure 2B). *App*^NL–G–F/E4^ females were lighter than WT females (*p* = 0.0032, Dunnett’s).

We also analyzed the data as a 2-way ANOVA with sex and donor genotype as factors. In recipient mice, there was an effect of sex (*F* (1, 54) = 62.878, *p* < 0.001), with females lighter than males. There was no effect of donor genotype. There was also an interaction between sex and donor genotype (*F* (2, 54) = 3.886, *p* = 0.026).

In donor mice, there was an effect of sex (*F* (1, 72) = 150.117, *p* < 0.001), with females being lighter than males. There was no effect of genotype. There was also an interaction between sex and genotype (*F* (2, 72) = 5.743, *p* = 0.005).

### Y Maze

**FIGURE 3:**
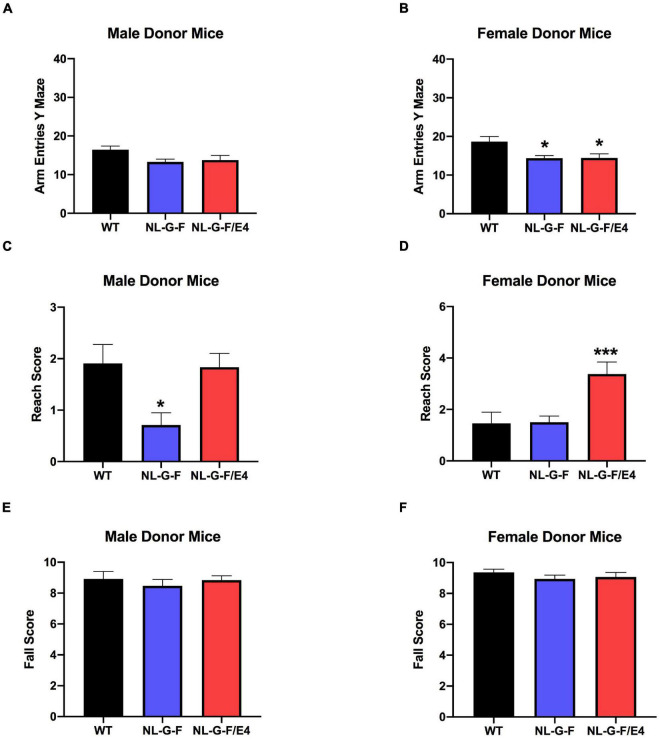
**(A)** There was no effect of genotype on arm entries in male donor mice. **(B)** There was an effect of genotype on arm entries in female donor mice (*F*(2,40) = 5.121, *p* = 0.0105). There were less arm entries in *App*^NL–G–F^ (*p* = 0.0119) and *App*^NL–G–F/E4^ (*p* = 0.0132, Dunnett’s) than WT female donor mice. *p < 0.05, Dunnett’s. **(C)** There was an effect of genotype on reach scores of male donor mice (*F*(2,49) = 5.341, *p* = 0.0047). Reach scores were lower in *App*^NL–G–F^ than WT mice. *p = 0.0217, Dunnett’s. **(D)**. There was an effect of genotype on reach scores of females (*F*(2,40) = 8.145, *p* = 0.0011) **(D)** donor mice. Reach scores were higher in *App*^NL–G–F/E4^ than WT mice. *p = 0.0039, Dunnett’s. **(E)** There was no effect of genotype on fall scores of male donor mice. **(F)** There was no effect of genotype on fall scores of female donor mice.

There was no effect of genotype on spontaneous alternation in male recipient mice (Figure 1C). There was no effect of genotype on spontaneous alternation in female recipient mice, but there was a trend toward a higher percent spontaneous alternation in *App*^NL–G–F/E4^ than WT females (*p* = 0.09, Dunnett’s). Arm entries were analyzed as activity measure in the Y maze. There was no effect of genotype on arm entries in males (Supplementary Figure 1A) or females (Supplementary Figure 1B). There was no effect of genotype on spontaneous alternation in male donor mice (Figure 2C). However, there was an effect of genotype on spontaneous alternation in female donor mice (*F* (2, 40) = 6.136, *p* = 0.0047), with a lower percent spontaneous alternation in *App*^NL–G–F^ than WT female donor mice (*p* = 0.0163, Dunnett’s). There was no effect of genotype on arm entries in male donor mice (Figure 3A). There was an effect of genotype on arm entries in female donor mice (*F* (2, 40) = 5.121, *p* = 0.0105), with less arm entries in *App*^NL–G–F^ (*p* = 0.0119, Dunnett’s) and *App*^NL–G–F/E4^ (*p* = 0.0132, Dunnett’s) than WT female donor mice (Figure 3B).

We also analyzed the data as a 2-way ANOVA with sex and donor genotype as factors. In recipient mice, in both recipient and donor mice, there was no effect of sex or donor genotype, and no interaction between the two on spontaneous alternation. For arm entries, there was no effect of sex or donor genotype in recipient mice.

In donor mice, there was no effect of sex, but an effect of genotype (*F* (2, 89) = 6.010, *p* = 0.004). WT mice had more entries than *App*^NL–G–F^ (*p* = 0.005) and *App*^NL–G–F/E4^ (*p* = 0.007) mice. There was no interaction between sex and genotype in donor mice for arm entries.

### Wire Hang

There was no effect of genotype on reach or fall scores of male (Supplementary Figures 1C,E) or female (Supplementary Figures 1D,F) recipient mice. However, there was an effect of genotype on reach scores of male (*F* (2, 49) = 5.341, *p* = 0.0047) (Figure 3C) and female (*F*(2,40) = 8.145, *p* = 0.0011) (Figure 3D) donor mice. In male donor mice, reach scores were lower in *App*^NL–G–F^ than WT mice (*p* = 0.0217, Dunnett’s). In female donor mice, reach scores were higher in *App*^NL–G–F/E4^ than WT mice (*p* = 0.0039, Dunnett’s). There was no effect of genotype on fall scores of male (Figure 3E) or female (Figure 3F) donor mice.

We also analyzed the data as a 2-way ANOVA with sex and donor genotype as factors. In recipient mice, there was an effect of sex on the fall score (*F* (1, 54) = 6.091, *p* = 0.017) and the reach score (*F* (1, 54) = 10.926, *p* = 0.002), with females having higher scores for both. There was no effect of transplant genotype and no interaction between transplant genotype and sex on the fall or reach score in recipient mice.

In donor mice, there was no effect of sex or genotype and no interaction between the two for fall score. For reach score, there was an effect of sex (*F* (1, 89) = 4.887, *p* = 0.030). Females had higher reach scores than males. There was also an effect of genotype (*F* (2, 89) = 11.490, *p* < 0.001). *APP^NLGF/E4^* mice had higher scores than *APP*^NLGF^ mice (*p* < 0.001). There was also an interaction between sex and genotype (*F* (2, 89) = 3.919, *p* = 0.023).

### Open Field and Novel Object Recognition

**FIGURE 4:**
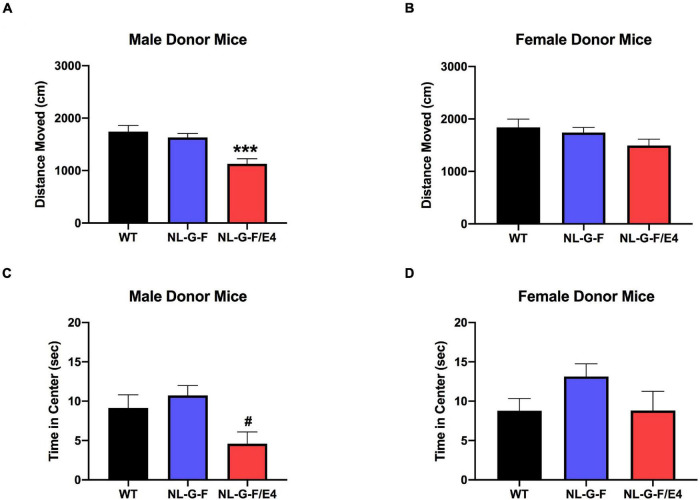
**(A)** There was an effect of genotype on activity levels in male donor mice (*F*(2,49) = 11.68, *p* < 0.0001). Activity levels were lower in *App*^NL–G–F/E4^ than WT mice ***p = 0.0003, Dunnett’s. **(B)** There was no effect of genotype on activity levels in female donor mice. **(C)** There was a trend toward less time spent in the center in *App*^NL–G–F/E4^ than WT male donor mice. ^#^*p* = 0.0938, Dunnett’s. **(D)** There was no effect of genotype on time spent in the center of the open field in female donor mice.

There was no effect of genotype on activity levels or time spent in the center of the open field in male (Supplementary Figures 2A,C) or female (Supplementary Figures 2B,D) recipient mice. There was an effect of genotype on activity levels in male donor mice (*F*(2,49) = 11.68, *p* < 0.0001), with lower activity levels in *App*^NL–G–F/E4^ than WT mice (*p* = 0.0003, Dunnett’s) (Figure 4A). There was no effect of genotype on activity levels in female donor mice (Figure 4B). There was an effect of genotype on percent time spent in the center of the open field in male donor mice (*F*(2,49) = 5.059, *p* = 0.0101), with a trend toward less time spent in the center in *App*^NL–G–F/E4^ than WT mice (*p* = 0.0938, Dunnett’s) (Figure 4C). In contrast, there was no effect of genotype on time spent in the center of the open field in female donor mice. The locomotion ratio in the center was also calculated for all mice by dividing the distance moved in the center by the total distance moved, and multiplying that number by 100. In both recipient and donor mice, there was no effect of sex or genotype, and no interaction between the two for center locomotion ratio.

When object recognition was analyzed in male recipient mice, there was a trend toward preferential exploration of the novel object in WT (*t* = 1.526, *p* = 0.0854 (*t*-test)) and preferential exploration of the novel object in *App*^NL–G–F/E4^ mice (*t* = 3.447, *p* = 0.0037 (*t*-test)) but *App*^NL–G–F^ mice showed impaired object recognition (Figure 1E). In female recipient mice, WT (*t* = 4.094, *p* = 0.0014 (*t*-test)) and *App*^NL–G–F/E4^ (*t* = 2.278, *p* = 0.0230 (*t*-test)) mice spent more time exploring the novel than familiar object and there was a trend toward *App*^NL–G–F^ mice exploring the novel object more than the familiar one (*t* = 1.657, *p* = 0.0681 (*t*-test)). We also compared the percent time spent with the novel object to chance (50%), and the results obtained were similar to the analysis presented here, with the percent time with the familiar object as the comparison.

We also analyzed the data as a 2-way ANOVA with sex and donor genotype as factors. In recipient mice, there was an effect of sex on distance moved on the first open field day (*F*(1,54) = 4.101, *p* = 0.048), with females moving more than males. There was no effect of donor genotype and no interaction between sex and donor genotype. There was no effect of sex or donor genotype and no interaction between the two for time spent in the center of the open field on day 1 in recipient mice.

In donor mice, there was an effect of sex on distance moved on the first open field day (*F*(1,89) = 10.905, *p* = 0.040), with females moving more than males. There was also an effect of genotype (*F*(2,89) = 4.887, *p* < 0.001). *APP*^NL–G–F^ mice moved more than *APP*^NL–G–F/E4^ mice (*p* < 0.001). WT mice moved more than *APP*^NL–G–F/E4^ mice (*p* < 0.001). There was no interaction between sex and genotype for distance moved in donor mice. For time in the center of the open field on day 1, there was no effect of sex, but there was an effect of genotype (*F*(2,89) = 4.995, *p* = 0.009). *APP*^NL–G–F^ mice spent more time in center than *App*^NL–G–F/E4^ mice (*p* = 0.003). There was no interaction between sex and genotype for time spent in the center in donor mice.

### Spatial Y Maze

There was no overall effect of genotype on the percent entries into the novel arm in the Y maze in recipient male mice but there was a trend toward a higher percent entries of *App*^NL–G–F/E4^ than WT mice into the novel arm of the Y maze (*p* = 0.0985 (Dunnett’s)) (Figure 1G). There was no effect of genotype on the percent entries into the novel arm in the Y maze in recipient female mice (Figure 1H). There was no effect of genotype on percent time recipient males or females spent in the novel arm of the Y maze.

We also analyzed the data as a 2-way ANOVA with sex and donor genotype as factors. In recipient mice, there was no effect of sex or donor genotype, and no interaction between the two, on the percent entries into the novel arm or the percent time in the novel arm.

### Cortical Insoluble Human Aβ40 Levels

**FIGURE 5:**
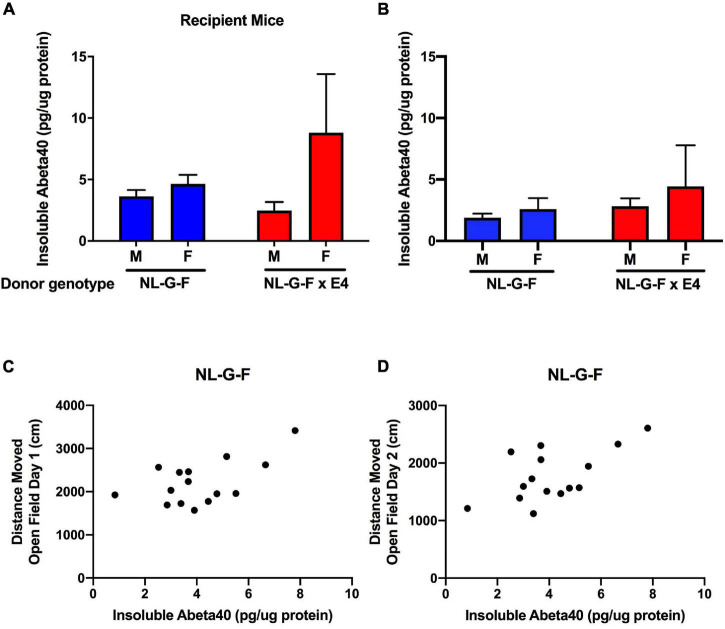
**(A)** Insoluble cortical Aβ40 levels in *App*^NL–G–F^ and *App*^NL–G–F/E4^ recipient mice. **(B)** Insoluble cortical Aβ40 levels in *App*^NL–G–F^ and *App*^NL–G–F/E4^ donor mice. **(C)** In *App*^NL–G–F^ recipient mice, cortical insoluble Aβ40 levels were positively correlated with activity levels on the first day of open field testing (*r* = 0.5629, *p* = 0.028, 15 data points, Pearson correlation). **(D)** In *App*^NL–G–F^ recipient mice, cortical insoluble Aβ40 levels also were positively correlated with activity levels on the second day of open field testing (*r* = 0.5949, *p* = 0.0193, 15 data points, Pearson correlation).

Cortical tissues from *App*^NL–G–F^ and *App*^NL–G–F/E4^ recipient and donor mice were processed to analyze soluble and insoluble Aβ40 and Aβ42 levels. In *App*^NL–G–F^ and *App*^NL–G–F/E4^ recipient mice, no soluble Aβ40 or Aβ42 levels were detected. Insoluble Aβ42 levels were only detected in 2 out of 19 *App*^NL–G–F^ recipient mice (2.7 and 17.8 pg/μg protein) and 1 out of 16 *App*^NL–G–F/E4^ recipient mice. However, insoluble Aβ40 levels were consistently detected in *App*^NL–G–F^ and *App*^NL–G–F/E4^ recipient mice (Figure 5A). There was no sex or genotype difference in insoluble *Aβ40* levels. The cortical insoluble Aβ40 levels in the recipient mice were remarkable considering the cortical insoluble Aβ40 levels in the genotype-matched donor mice (Figure 5B). In *App*^NL–G–F^ mice, the cortical insoluble Aβ40 levels were higher in recipient than donor mice (*t* = 2,697, *p* = 0.0139, 2-tailed unpaired t-test). This was genotype dependent and not seen in *App*^NL–G–F/E4^ mice.

Next, we assessed whether in recipient mice cortical insoluble human Aβ40 levels related to the behavioral measures. In *App*^NL–G–F^ recipient mice, cortical insoluble Aβ40 levels were positively correlated with activity levels on the first (*r* = 0.5629, *p* = 0.028, 15 data points, Pearson correlation, Figure 5C) and second (*r* = 0.5949, *p* = 0.0193, 15 data points, Pearson correlation, Figure 5D) day of open field testing. This relationship was genotype-dependent and not seen in *App*^NL–G–F/E4^ recipient mice.

### Gut Microbiome

**FIGURE 6:**
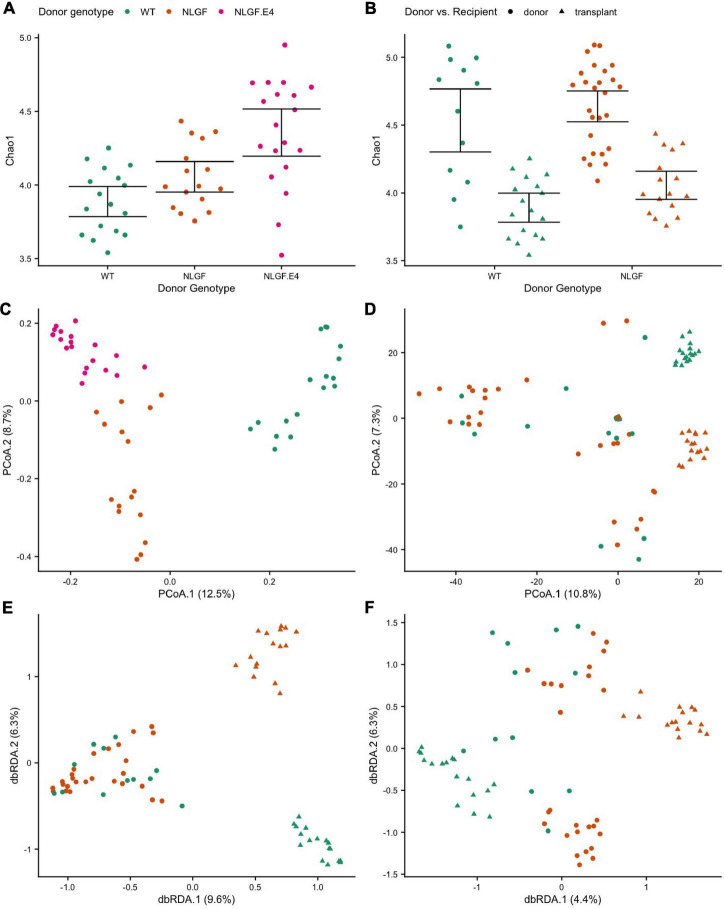
Alpha- and beta-diversity scores by donor genotype and/or transplant status. **(A)** Chao1 index scores by donor genotype for recipients of microbiome transplants showing increased diversity for microbiomes transplanted from *App*^NL–G–F/E4^ mice donors compared to transplants from WT donors. **(B)** Chao1 index scores by donor genotype and donor vs recipient samples showing decreased diversity in transplanted microbiomes compared to donor microbiomes, regardless of genotype. **(C)** Principal Coordinate Analysis (PCoA) ordination of differences in microbiome sample composition according to Canberra distances by donor genotype for recipient samples showing that transplanted microbiomes cluster by donor genotype. **(D)** PCoA ordination of Canberra distances by donor genotype and donor vs recipient samples showing that microbiomes cluster by transplant status (donor or recipient) and donor genotype. **(E)** Distance-based Redundancy Analysis (dbRDA) ordination of Canberra distances constrained to maximize variance by donor genotype and donor vs recipient status, which highlights the small differences in composition between donor microbiome genotypes, but much larger differences between microbiome of donor genotypes in the recipient mice. **(F)** dbRDA ordination of Canberra distances constrained by donor genotype after accounting for variance explained by donor vs. recipient status, which highlights the significant effects of donor genotype on the recipient microbiome composition even after accounting for the effects of transplantation.

Overall, gut microbiome diversity and composition associated with the genotype of the donor and whether a mouse was a donor or a recipient. Moreover, we observed associations between measures of behavioral performance and the microbiomes in transplant recipient mice. Specifically, for mice that were recipients of microbiome transplants, donor genotype significantly associated with the alpha-diversity of their microbiomes. Transplanted microbiomes from WT donors had, on average, the lowest diversity, while microbiomes transplanted from *App*^NL–G–F/E4^ donors had, on average the highest diversity (Figure 6A). That said, transplanted microbiomes generally manifested lower alpha-diversity than that of the donor communities (Figure 6B), indicating that transplantation elicits specific effects on the gut microbiome. Donor genotype also significantly predicted microbiome composition of transplanted microbiomes in recipient mice (Figure 6C), indicating that donor genotype elicits a strong and specific effect on the composition of the gut microbiome. Moreover, transplanted microbiomes from a particular donor genotype were also much more similar to one another in composition relative to the interindividual variation observed among the respective donor microbiomes while also being more distinct across donor genotypes than observed for donor microbiome samples (Figure 6D). That said, donor genotype was a significant predictor of microbiome composition for both donor and recipient communities, even after the effects of transplant status was statistically controlled for Figures 6D–F.

**FIGURE 7:**
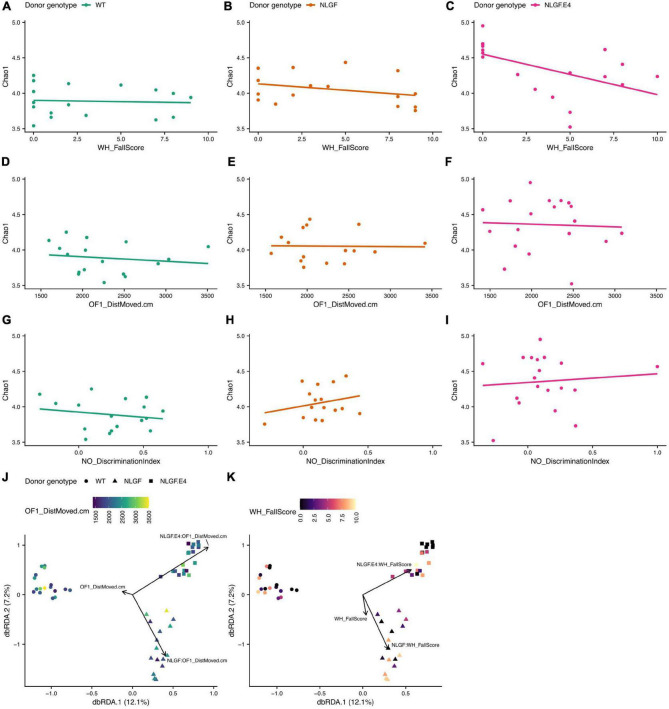
Alpha- and beta-diversity scores of recipient samples by behavior scores and donor genotype. **(A–C)** Chao1 index scores by WH_FallScore for samples with microbiomes transplanted from WT **(A)**, *App*^NL–G–F^, **(B)**, or *App*^NL–G–F/E4^ **(C)** mice. **(D–F)** Chao1 index scores by OF1-DistMoved.cm for recipients of microbiome transplants from each donor genotype, same order as **(A–C)**. **(G–I)** Chao1 index scores by NO_DiscriminationIndex for recipients of microbiome transplants from each donor genotype, same order as **(A–C)**. **(J)** dbRDA ordination of Canberra distances of differences in composition between samples. The shape of the point indicates the donor genotype and the color of the point indicates the OF1_DistMoved.cm score for that sample. The black arrows indicate that the direction of greatest change for the OF1_DistMoved.cm score interacts significantly with the genotype of the donor that provided the microbiome transplant. **(K)** dbRDA ordination [exact same ordination as in J] of Canberra distances of differences in composition between samples. The shape of the point indicates the donor genotype and the color of the point indicates the WH_FallScore for that sample. The black arrows indicate that the direction of greatest change for the WH_FallScore score interacts significantly with the genotype of the donor that provided the microbiome transplant.

The alpha diversity of the gut microbiome of recipient mice also associated with several behavioral scores in a donor genotype dependent manner. In particular, performance in the wire hang test (WH_FallScore, Figures 7A–C), distance moved on the first day of open field testing (OF1_DistMoved.cm, Figures 7D–F), and object recognition (NO_DiscriminationIndex, Figures 7G–I) had different associations with the Chao1 diversity index depending on the donor genotype. For example, the relationship between WH_FallScore and Chao1 in mice that received WT microbiomes was rather flat and not significantly different from a slope of zero. However, this same relationship in mice that received microbiomes from *App*^NL–G–F/E4^ mice was significantly negative, meaning that mice with higher scores in the wire hang test tended to have lower Chao1 scores. Similarly, two behavior scores – distance moved on the first day of open field testing (OF1_DistMoved.cm) and performance in the wire hang test (WH_FallScore) – significantly predicted microbiome composition in recipient mice, but the compositions that associated with these measures differed as a function of donor genotype (Figures 7J,K).

## Discussion

The results of this study show that it is feasible to behaviorally test germ-free mice inside a biosafety cabinet. The activity and anxiety levels in the open field and the percent spontaneous alternations in the Y maze were comparable in range in donor and recipient mice. This is remarkable based on the anticipated more enclosed setting of a biosafety cabinet and the smaller size of Y maze used in the biosafety cabinet. Perhaps related to the size of the Y mazes, arm entries, a measure of activity in the Y maze, was higher in recipient than donor mice. Fall scores in the wire hang test were lower in recipient than donor mice, while reach scores were comparable. The results of this study also show that stool from 6-month-old *App*^NL–G–F^ mice or *App*^NL–G–F^ crossed with human apoE4 targeted replacement mice is sufficient to induce behavioral phenotypes in 4-5 month-old germ-free C57BL/6J mice 4 weeks following inoculation. This is consistent with the observations that the gut microbiome can communicate with the CNS via the gut-brain axis, and affect stress-related behaviors, anxiety, and depression (Foster and McVey Neufeld, 2013; Kelly et al., 2015; Allen et al., 2017) and that behavioral phenotypes can be transferred using fecal microbiota transplantation (Bercik et al., 2011; Collins et al., 2013; De Palma et al., 2017). However, the host genotype was critical in modulating the pattern of induced behavioral phenotypes as compared to those seen in the genotype- and sex-match donor mice (Table 1). Our gut microbiome analyses revealed that gut microbiome diversity and composition associate with behavioral measures and that genotype, and to a lesser extent sex, modifies this relationship. In recipient mice, the relationship between cortical insoluble human Aβ40 levels and activity levels in the open field was also genotype-dependent. In *App*^NL–G–F,^ but not in *App*^NL–G–F/E4^, recipient mice, cortical insoluble Aβ40 levels were positively correlated with activity levels on in the open field. Therefore, genotypes and sexes of the donor and recipient need to be considered for developing therapeutic strategies with fecal implants in AD.

In recipient males, body weights were lower in *App*^NL–G–F^ and *App*^NL–G–F/E4^ mice than WT mice, while no genotype differences were seen in donor mice. As loss of body mass index is associated with increased AD risk, and this effect seems more profound in females than males (Kang et al., 2021), these data suggest that the host environment, such as genetic differences in metabolic pathways, in the *App*^NL–G–F^ and *App*^NL–G–F/E4^ donor males might protect them against a loss in body weight. This protective effect is sex-dependent as the opposite was seen in females. In recipient females, body weights were higher in *App*^NL–G–F/E4^ mice than WT mice, while lower body were seen in *App*^NL–G–F/E4^ than WT donor mice. Similarly, reduced hippocampus-dependent spontaneous alternation was seen in *App*^NL–G–F^ donor but not recipient female mice. These data highlight the critical role of the host genotype environment in modulating those effects.

*App*^NL–G–F/E4^ recipient males, but not females, showed impaired object recognition. In contrast, there was no genotype difference in percent spontaneous alternation and there was a trend toward a higher percent entries into the novel arm of the Y maze in *App*^NL–G–F/E4^ recipient males. Considering that both the object recognition test and novel arm Y maze test both involved a 24-hr interval between training/learning and memory testing, these data illustrate that those hippocampus-dependent tests assess different abilities and highlight the importance of including several hippocampus-dependent tests in assessing cognitive phenotypes. *App*^NL–G–F/E4^ recipient males, but not females, showed impaired object recognition. However, there was a trend toward an increased percent entries into the novel arm of the Y maze in *App*^NL–G–F/E4^ recipient males, but not females. These results are consistent with the novel arm Y maze test being more spatial navigational biased toward better performance of males under the test conditions (*i.e.*, landmarks/cues on the maze) used (Moffat et al., 1998; Sandstrom et al., 1998), while the object recognition test is biased toward better performance in women and female rats and in female mice when the novel and familiar objects are more similar to each other (Bettis and Jacob, 2012).

A limitation of the current study is that the recipient mice were slightly younger than the recipient mice. It is conceivable that additional phenotypes might have been revealed in older recipient mice. In addition, additional phenotypes in recipient mice might have been revealed and a similar pattern in phenotypes between recipient and donor mice if the recipient mice would have been genotype-matched. A subset of melanoma patients resistant to immunotherapy therapy with anti-PD1 became sensitive to immunotherapy following fecal microbiota transplantation from responsive melanoma patients, indicating that in that context the genotype of the host might be less critical (Davar and Al, 2021). This might be less critical because only a subset of melanomal patients that were resistant became sensitive. The genotype of the host did not seem critical either in inducing PD pathology in α-synuclein-overexpressing mice receiving fecal transplants from PD patients compared to those receiving fecal transplants from healthy human donors ^5^. From a translational perspective, increased understanding of the role of the host genotype and host environment in modulating the phenotypes will be important and increased efforts for studies along those lines are warranted. Regardless, these data demonstrate that the gut-brain axis likely plays an important role in AD and related neurodegenerative conditions.

In summary, behavioral testing of fecal transplanted germ-free mice in a biosafety cabinet is feasible and stool from *App*^NL–G–F^ and *App*^NL–G–F/E4^ mice is sufficient to induce behavioral phenotypes in germ-free C57BL/6J mice 4 weeks following inoculation. In addition, gut microbiome diversity and composition associate with behavioral measures, and genotype modifies this relationship. Moreover, the relationship between cortical insoluble human Aβ40 levels and activity levels in the open field is also genotype-dependent. The detectable cortical insoluble Aβ40 levels in the recipient mice are consistent with the ability of Aβ40 to induce amyloid aggregation in WT mouse brain (De Mena et al., 2020). The cross reactivity of the antibody used in the human Aβ40 ELISA is 0.5%. Therefore, we cannot exclude that some or even all of the cortical insoluble Aβ40 detected is of murine origin. Future efforts are warranted to explore these distinct scenarios.

## Data Availability Statement

The raw data supporting the conclusions of this article will be made available by the authors, without undue reservation.

## Ethics Statement

The animal study was reviewed and approved by OHSU and OSU IACUC Committees.

## Author Contributions

PK and JR designed the study. NS supervised the fecal transplantation and monitoring of the mice in the germ-free facility. PK and SH behaviorally tested the mice. PK and JR analyzed the data and prepared the figures with the behavioral data. JR and SH processed the cortical samples for the Aβ ELISAs and analyzed the related data. KS and TS analyzed the gut microbiome data and the relationship with the behavioral measures and generated the related figures. All authors contribute to and reviewed the manuscript.

## Conflict of Interest

The authors declare that the research was conducted in the absence of any commercial or financial relationships that could be construed as a potential conflict of interest.

## Publisher’s Note

All claims expressed in this article are solely those of the authors and do not necessarily represent those of their affiliated organizations, or those of the publisher, the editors and the reviewers. Any product that may be evaluated in this article, or claim that may be made by its manufacturer, is not guaranteed or endorsed by the publisher.
