# Supplementary figures and images for: Fecal Implants From AppNL–G–F and AppNL–G–F/E4 Donor Mice Sufficient to Induce Behavioral Phenotypes in Germ-Free Mice
Source: Front Behav Neurosci. 2022 Feb 8;16:791128. doi: 10.3389/fnbeh.2022.791128 (PMC8860839; doi:10.3389/fnbeh.2022.791128)

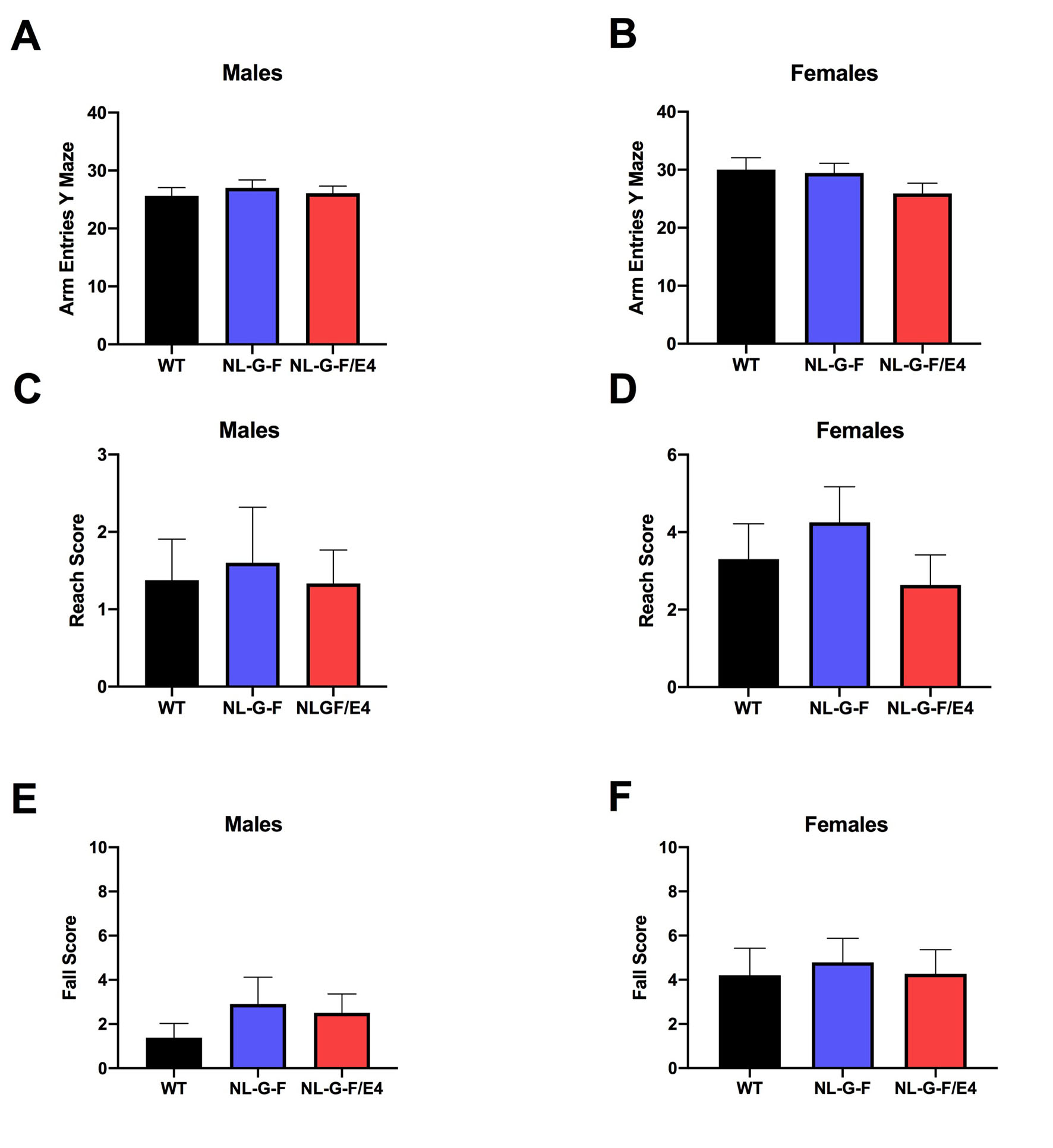

Supplement: Supplementary file 1 [file Image_1.JPEG]

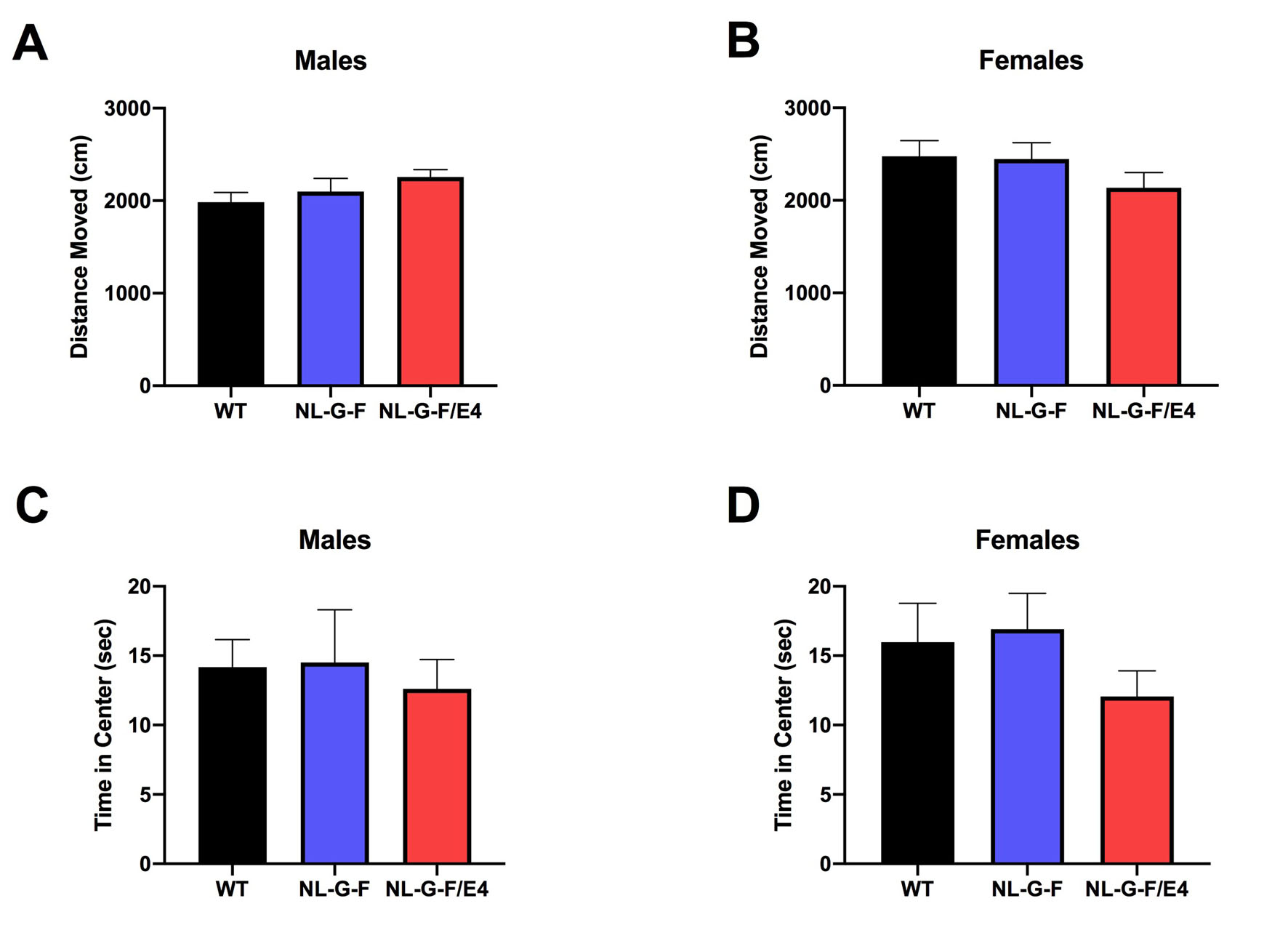

Supplement: Supplementary file 2 [file Image_2.JPEG]
